# Extreme and Cyclical Blood Pressure Elevation in a Pheochromocytoma Hypertensive Crisis

**DOI:** 10.1155/2018/4073536

**Published:** 2018-07-15

**Authors:** V. Larouche, N. Garfield, E. Mitmaker

**Affiliations:** ^1^Adult Endocrinology and Metabolism Training Program, McGill University, Montréal, QC, Canada; ^2^Division of Endocrinology, McGill University Health Centre, Montréal, QC, Canada; ^3^Division of General Surgery, McGill University Health Centre, Montréal, QC, Canada

## Abstract

Pheochromocytomas are rare adrenal neoplasms characterized by excess secretion of catecholamines. We describe the case of a 65-year-old man, known for hypertension, with no family history of hereditary pheochromocytoma syndromes. He reported a two-year history of flushing, systolic blood pressure surges to 200 mmHg, headaches, tremors, and syncope. His initial workup revealed elevated 24h urine catecholamines and metanephrines. An adrenal MRI in March 2017 showed a large 7.6 cm heterogeneous right adrenal lesion. Given orthostatic hypotension, his final preoperative dose was limited to a low dose of terazosin and metoprolol. In the operating room, shortly after intubation and Foley insertion, his blood pressure rose to 350 mmHg. Surgery was cancelled and he was admitted to the intensive care unit, where intravenous phentolamine, nitroprusside, and nicardipine were started. His systolic blood pressure would oscillate between 60 mmHg and 350 mmHg at 2-3 minutes' intervals. After 3 days, he was weaned off intravenous medications. His oral medications were uptitrated to high doses of phenoxybenzamine, metoprolol, and nifedipine. Three weeks later, he underwent successful open right adrenalectomy. This case outlines the importance of preoperative preparation of pheochromocytomas and raises the question if phenoxybenzamine is the alpha-blocker of choice for larger tumours with significant hormonal secretion.

## 1. Introduction

Catecholamine-secreting tumours that arise from chromaffin cells of the adrenal medulla and the sympathetic ganglia are referred to as pheochromocytomas and paragangliomas. Their annual incidence is 0.8 per 100,000 person-years. They are most common in the fourth to fifth decade and are equally common in men and women. The classic triad of symptoms consists of episodic headaches, sweating, and tachycardia. Most patients do not have the three classic symptoms. The diagnosis of pheochromocytoma is made based upon biochemical confirmation of catecholamine hypersecretion with 24h urine collection of metanephrines or plasma-free metanephrines followed by imaging studies such as CT Scan or MRI of the adrenals and possibly functional imaging with nuclear medicine modalities. The mainstay of management of pheochromocytoma is surgical excision of the tumour with careful preoperative preparation which includes volume expansion, alpha-adrenergic blockade first, followed by beta-adrenergic blockade. This case highlights the rare event of an extreme and cyclical blood pressure pattern in the context of a pheochromocytoma hypertensive crisis.

## 2. Case Presentation

We report the case of a 65-year-old man known for hypertension, cholelithiasis, and panic disorder with no personal or family history of pheochromocytoma, paraganglioma, Multiple Endocrine Neoplasia Type 2 syndrome, Von Hippel Lindau syndrome, Neurofibromatosis Type 1, or Succinyl Dehydrogenase mutations. He is a past smoker who quit 5 years prior to presentation and cumulated a 20-pack-year smoking history with no history of dyslipidemia or diabetes. The patient described a two-year history of frequent episodes of flushing, diaphoresis, systolic blood pressure surges up to 200 mmHg, loss of vision, headaches, palpitations, and tremors. He also complained of more frequent episodes of presyncope up to 6 times a day in the few weeks prior to seeking medical attention. The patient denied pallor, weight loss, weakness, or abdominal pain. His blood pressure was episodically elevated with only a moderately elevated baseline blood pressure. His only antihypertensive therapy at his first visit to our Endocrinology clinic was terazosin 1 mg once daily with only partial relief of his paroxysmal symptoms.

The patient was initially diagnosed with panic disorder and treated with cognitive-behavioural therapy. On physical exam, the patient's weight was 92 kg, his height was 1.77 m, and his BMI was 29.4 kg/m2. His blood pressure was 168/100 mmHg; his heart rate was regular between 90 and 100 bpm. His abdominal exam, however, revealed an obese nontender abdomen with a palpable right-sided suprarenal mass of 6-7 cm diameter, which was soft and mobile.

Two 24h urine collections for metanephrines and catecholamines were performed (c.f.  Table 1) and confirmed hypersecretion. No plasma aldosterone or renin levels were drawn, and no Cushing syndrome screening test was performed.

**Figure 1:**
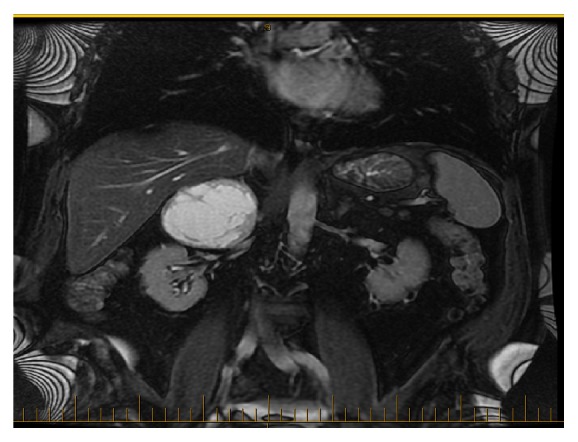
MRI adrenals.

An MRI of the adrenals (c.f.  Figure 1) reported a large right adrenal mass measuring 7.6 x 7.6 x 7.2 cm with T2 hyperintensity centrally and no loss of signal in T1. It was reported as highly suspicious for pheochromocytoma with a normal left adrenal gland, liver, and pancreas and no evidence of metastasis. Of note, the patient was not known for any underlying cardiac arrhythmia and had a normal baseline electrocardiogram in sinus rhythm at 95 bpm. No echocardiogram was ordered preoperatively.

The patient was referred to a urologist at an academic centre. He was then seen by Endocrinology one month prior to surgery. At his first visit to our clinic, he was counselled to have a high-salt diet and oral hydration was encouraged. The dose of terazosin was increased to 1 mg po bid x 1 week and then 2 mg po bid. Chromogranin A was elevated: 274 ng/mL (N < 82 ng/mL). An MIBG Scan was ordered, but the radiotracer was unavailable.

The patient was closely followed up by phone every week. Terazosin was increased to 2 mg po bid and metoprolol was added and increased to a maximal dose of 25 mg tid until he demonstrated orthostatic hypotension. The patient was admitted one day prior to surgery with a well-controlled blood pressure of 112/70 mmHg.

In the operating room, shortly after intubation and Foley catheter insertion and prior to surgical incision, the patient's blood pressure rose to 350/180 mmHg without improvement despite intravenous phentolamine boluses. Given the inability to control the patient's labile blood pressure, a decision was made to abort the operation and transfer the patient to the intensive care unit while remaining intubated.

**Figure 2:**
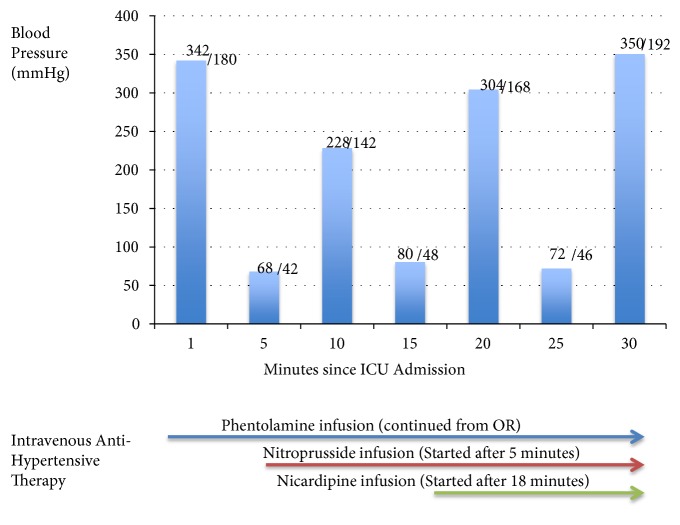
Blood pressure after admission to ICU.

In the intensive care unit, the patient required massive doses of intravenous phentolamine, nitroprusside, and nicardipine as well as intravenous hydration, as these are the main options for management of a pheochromocytoma hypertensive crisis as per current clinical practice guidelines [1]. Interestingly, his blood pressure would oscillate between 60/34 and 350/186 mmHg within a matter of 2-3 minutes in a cyclical pattern (c.f.  Figure 2). A nasogastric tube was inserted, and the patient was started on phenoxybenzamine per tube. After 72 hours in ICU, he was weaned off intravenous antihypertensives and sedatives and extubated.

He was transferred back to the surgical ward, while gradually having his blood pressure medications uptitrated to phenoxybenzamine 120 mg po bid, metoprolol 100 mg po bid, and nifedipine XL 60 mg once daily. His blood pressure was then well controlled. The patient underwent successful open right adrenalectomy three weeks later. He was hypotensive intraoperatively, requiring vasopressors. He had an uncomplicated postoperative course.

Further investigations in hospital during his stay included free plasma normetanephrine 14.44 nmol/L (N<1.2 nmol/L) and metanephrine 6.09 nmol/L (N< 0.48 nmol/L), a negative cerebral CT scan, and an Octreoscan showing no site of extra-adrenal uptake. An Octreoscan was performed as a surrogate functional imaging modality given the unavailability of MIBG (recommended functional imaging modality) radiotracer at our centre around the time of this patient's admission. Pathology confirmed an 8 cm right adrenal pheochromocytoma without angioinvasion, extra-adrenal extension, or necrosis.

One month postoperatively, the patient was seen in the Endocrinology clinic and reported feeling overall well with no documented hypertension. He stopped all antihypertensive medications and had no palpitations, diaphoresis, flushing, headaches, or other symptoms of catecholamine excess. His 24h urine metanephrines and catecholamines normalized during follow-up (c.f.  Table 1). The patient has not undergone genetic analysis during follow-up.

Unfortunately, the patient sustained a ST-elevation myocardial infarction three months postoperatively, requiring urgent percutaneous coronary intervention and stent placement. Unfortunately, he was treated at another hospital and results from his coronary angiogram were not available to authors. He survived this acute cardiac event and continues to be followed closely by Cardiology. His longstanding elevated blood pressure secondary to his pheochromocytoma and his prior smoking history were significant risk factors for his myocardial infarction.

## 3. Discussion

In summary, our patient is a 65-year-old man who developed a hypertensive crisis with extreme and cyclical blood pressure due to insufficient preoperative alpha- and beta-blockade in planning for an open adrenalectomy to resect a 7.6 cm right adrenal pheochromocytoma.

A Medline search from 1980 until now using the keywords “pheochromocytoma”, “cyclic”, ”blood pressure”, and ”hypertension” revealed 8 similar cases of pheochromocytoma with recurrent oscillations between hypertension and hypotension.

As outlined in Table 2, Ganguly and colleagues [2] described a 67-year-old man with a 6 cm right-sided pheochromocytoma admitted to the coronary care unit with chest pain, adrenergic symptoms, bradycardia, and a systolic blood pressure varying between 60 and 240 mmHg every 5-10 minutes. Similarly, Guzik and colleagues [3] described a 52-year-old woman with a 10 cm right adrenal pheochromocytoma, norepinephrine predominant, who presented with cyclical blood pressure elevation up to 316 mmHg and decreased down to 50 mmHg.

Furthermore, Kobal and colleagues [4] reported the case of a 52-year-old woman with a 2.5 cm right adrenal pheochromocytoma, metanephrine predominant, who presented to the emergency room with an 8-hour history of chest and abdominal pain. She also had significant systolic blood pressure surges, up to 265 mmHg and down to 30 mmHg. Ionescu and colleagues [5] described a 47-year-old woman with a 4.2 cm right-sided adrenal pheochromocytoma, epinephrine predominant, who developed rapid oscillations between hypertension (BP up to 344/170 mmHg) and hypotension (BP down to 52/34 mmHg) every 14 minutes in an electrophysiological study for palpitations, triggered by anesthetic agents.

Jindal and colleagues [6] reported the case of a 55-year-old man with a 5.2 cm right adrenal pheochromocytoma, multihormone secreting, who presented with cyclical blood pressure variations from 80/55 mmHg to 190/99 mmHg in the context of Takotsubo cardiomyopathy. Along the same lines, Murai and colleagues [7] described a 42-year-old man with a 4.2 cm right-sided pheochromocytoma, secreting mostly norepinephrine and epinephrine, who presented with acute chest pain and blood pressure readings varying between 70/50 mmHg and 160/100 mmHg at 15 minutes' intervals.

Oishi and colleagues [8] reported a 69-year-old man with a right adrenal pheochromocytoma, norepinephrine predominant, who presented with blood pressure fluctuations in 9-13 minutes' cycles. Moreover, Wenting and colleagues [9] reported an 18-year-old man with a norepinephrine predominant pheochromocytoma whose blood pressure varied by amplitudes up to 80 mmHg at three minutes' intervals during intra-arterial pressure monitoring.

Overall, cases included 5 males and 3 females aged 18-69, all with right-sided adrenal pheochromocytomas (when side mentioned) with a largest diameter between 2.5 and 10 cm, mostly epinephrine or norepinephrine predominant. [2–9]. For those cases where the outcome was described, all patients had a successful adrenalectomy. For those cases where the surgical approach was detailed, all surgeries were open as opposed to laparoscopic. Open approach is generally recommended for hemodynamically unstable patients. The patient described herein had a similar clinical presentation as the other reported cases.

In this case, our patient was preoperatively prepared with small doses of alpha-blockade and beta-blockade as per current guidelines [1]. Dose increase was limited by orthostatic hypotension. In retrospect, he was inadequately blocked and possibly volume contracted, as he developed a hypertensive crisis with cyclical blood pressure changes after anesthetic induction and Foley catheter insertion. Selective alpha-blockers are commonly used, as the nonselective alpha-blocker phenoxybenzamine, which remains the gold standard, is not easily accessible in Canada.

Few studies have compared nonselective versus selective alpha-blockers for preoperative management of pheochromocytoma. Both Zhu et al. [10] and Weingarten et al. [11] showed that preoperative preparation with selective alpha-blocker leads to higher intraoperative systolic BP but less arterial BP fluctuation compared to phenoxybenzamine. No clear size cut-off has been identified as a factor to choose nonselective over selective alpha-blocker.

Interestingly enough, Groeben et al. [12] conducted an observational case series where they compared haemodynamic conditions and perioperative complications of 110 patients with pheochromocytoma who receive preoperative alpha-blockade to 166 patients who did not. Their data showed a slightly lower mean maximal systolic arterial pressure (17 mmHg, p = 0.024) in those who received preoperative alpha-blockade with no difference in the incidence of excessive hypertensive episodes or major complications. Further studies are needed to identify the optimal preoperative pharmacological regimen for individual cases of pheochromocytoma.

## 4. Learning Points

- Preoperative preparation with alpha-blockers first and then with beta-blockers, when preparing a patient for an adrenalectomy due to a pheochromocytoma, is of utmost importance in order to decrease the risk of intraoperative hemodynamic instability.
- As per current guidelines, the nonselective alpha-blocker phenoxybenzamine remains the gold standard for preoperative preparation of a pheochromocytoma resection. However, no clear size cut-off or hormone level has been established in literature to guide clinicians to choose phenoxybenzamine over selective alpha-blockers.
- Pheochromocytoma hypertensive crisis is a medical emergency requiring intensive care unit admission and prompt management of the blood pressure with intravenous antihypertensives and fluid repletion.
- In catecholamine-mediated hypertensive crises, the blood pressure elevation can be extreme and cyclical in nature, varying between hypertensive surges and hypotensive episodes, therefore representing a clinical challenge.

## Figures and Tables

**Table 1:** Biochemical investigation.

24h urine collection for Metanephrines and Catecholamines	2016-12-26	2017-03-28	2017-07-13 (2 weeks post-op)	2017-10-12 (3 months post-op)	Normal Range
Epinephrine	219	642	Undet.	Undet.	0-110 nmol/d

Norepinephrine	782	1246	504	336	0-480 nmol/d

Dopamine	1368	1626	2519	1904	0-2620 nmol/d

Metanephrines	1718	3118	1869	Undet.	0-275 nmol/d

Normetanephrines	3478	5762	291	160	0-240 nmol/d

**Table 2:** Summary of similar cases.

Ref.	Age	Sex	Maximal Diameter (cm)	Side	Predominant Hormone	Blood Pressure Pattern	Clinical outcome
[1]	67	M	6.0	R	-	Cyclical Oscillations of sBP between 60-240 mmHg every 5-10 minutes	Successful right adrenalectomy.

[2]	52	F	10.0	R	NE	Cyclical Oscillations of sBP between 50-316 mmHg every 17 minutes	N/A

[3]	52	F	2.5	R	MN	Cyclical Oscillations of sBP between 30 and 265 mmHg every 15 minutes	Successful open right adrenalectomy.

[4]	47	F	4.2	R	EPI	Cyclical oscillations of BP from 52/34 to 344/170 mmHg every 14 minutes.	Successful open right adrenalectomy.

[5]	55	M	5.2	R	EPI/NE/DO	Cyclical oscillations of BP from 80/55 to 190/99 mmHg every 30 minutes	Successful open right adrenalectomy.

[6]	42	M	4.2	R	EPI/NE	Cyclical oscillations of BP from 70/50 to 160/100 mmHg every 15 minutes	Successful open right adrenalectomy.

[7]	69	M	-	R	NE	Cyclical oscillations of BP every 9-13 minutes	N/A

[8]	18	M	-	-	NE	Cyclical oscillations of BP every 3 minutes	N/A
